# Green Synthesis and Antibacterial Activity of Ag/Fe_2_O_3_ Nanocomposite Using *Buddleja lindleyana* Extract

**DOI:** 10.3390/bioengineering9090452

**Published:** 2022-09-08

**Authors:** Fatimah A. M. Al-Zahrani, Salem S. Salem, Huda A. Al-Ghamdi, Laila M. Nhari, Long Lin, Reda M. El-Shishtawy

**Affiliations:** 1 Chemistry Department, Faculty of Science, King Khalid University, Abha 61413, Saudi Arabia; 2 Botany and Microbiology Department, Faculty of Science, Al-Azhar University, Cairo 11884, Egypt; 3 Chemistry Department, Faculty of Science, University of Jeddah, Jeddah 21959, Saudi Arabia; 4 Colour Science, School of Chemistry, University of Leeds, Woodhouse Lane, Leeds LS2 9JT, UK; 5 Chemistry Department, Faculty of Science, King Abdulaziz University, Jeddah 21589, Saudi Arabia; 6 Dyeing, Printing and Textile Auxiliaries Department, Institute of Textile Research and Technology, National Research Centre, 33 EL Buhouth St., Dokki, Giza 12622, Egypt

**Keywords:** *Buddleja lindleyana*, Ag/Fe_2_O_3_, nanocomposite, phytosynthesis, antimicrobial activity

## Abstract

In the study reported in this manuscript, silver/iron oxide nanocomposites (Ag/Fe_2_O_3_) were phytosynthesized using the extract of *Buddleja lindleyana* via a green, economical and eco-friendly strategy. The biosynthesized Ag/Fe_2_O_3_ nanocomposites were characterized using UV-Vis spectrophotometry, FTIR, XRD, TEM, DLS and SEM-EDX analyses. The particulates showed a triangular and spherical morphology having sizes between 25 and 174 nm. FTIR studies on the nanoparticles showed functional groups corresponding to organic metabolites, which reduce and stabilize the Ag/Fe_2_O_3_ nanocomposite. The antimicrobial efficacy of the phytosynthesized Ag/Fe_2_O_3_ against bacterial pathogens was assessed. In addition, Ag/Fe_2_O_3_ exhibited broad spectrum activities against *B. subtilis*, *S. aureus*, *E. coli*, and *P. aeruginosa* with inhibition zones of 23.4 ± 0.75, 22.3 ± 0.57, 20.8 ± 1.6, and 19.5 ± 0.5 mm, respectively. The Ag/Fe_2_O_3_ composites obtained showed promising antibacterial action against human bacterial pathogens (*S. aureus*, *E. coli*, *B. subtilis* and *P. aeruginosa*), making them candidates for medical applications.

## 1. Introduction

In recent years, leaf extract-mediated biosynthesis of nanomaterials has been extensively studied [1,2,3,4]. Leaf extract-mediated synthesis of nanomaterials is more eco-friendly and economical than other methods of synthesis such as chemical reduction and physical methods [5,6,7]. Due to the growing demand for ecologically friendly material synthesis techniques, the biosynthesis of nanomaterials has gained attention as an emerging feature of the interface of nanotechnology and biotechnology [8,9,10,11,12,13,14,15,16,17]. The biosynthesis of inorganic materials, particularly metal nanoparticles, utilizing microbes and plants has received a lot of attention [18,19,20,21,22,23,24]. Nanosilver has many important applications [25,26,27,28]. Antimicrobial agents have been utilized with it [29,30]. The study reported in the first paper on green leaf cell extract solution-mediated synthesis of Ag/Fe_2_O_3_ hybrid nanoparticles successfully employed a green method to synthesize Fe_2_O_3_ as well as Ag/Fe_2_O_3_ nanoparticles. The synthesized nanoparticles showed excellent antibacterial, antifungal and anticancer activities [31]. Utilizing various reducing agents as well as no reducing agents, it was successful to produce nano-hybrid Ag/Fe_2_O_3_NPs depending on carboxymethyl–chitosan. Their fairly reversible magnetization curves show that γ-Fe_2_O_3_ NPs transition to a superparamagnetic state [32]. Antibacterial testing on nanocomposites revealed good antimicrobial action against both Gram-positive and Gram-negative bacteria, as well as good activity against yeast and *S. aureus* resistant strain [33]. The Ag/Fe_2_O_3_-Graphene oxide (GO) nanocomposites have shown superior long-term antibacterial capabilities against bacteria (Gram-negative and Gram-positive bacteria), demonstrating their particular potential as a promising long-term biocide with minimal environmental hazard. These abilities were compared to plain Ag nano and Ag/Fe_2_O_3_ [34]. The creation of Ag on magnetic-Fe_3_O_4_ NPs surfaces modified using *Stachys lavandulifolia* extract as a reducing and stabilizing magnetic agent was proposed as an effective and environmentally friendly method. Furthermore, the 4-nitrophynol reduction using the nanocomposite and its bactericidal activity was evaluated [35]. Nanocomposites such as Fe_3_O_4_ or Ag/Fe_2_O_3_ are covered by porous silica oxides serving as magnetic cores to carry anticancer drugs [36]. Nanomaterials which have magnetic behavior, such as Fe_3_O_4_ or Ag/ Fe_2_O_3_, are good candidates for superparamagnetism [37,38]. Magnetic resonance imagining (MRI) investigations also utilize iron oxide nanoparticles [39]. The combination of Ag and Fe_2_O_3_ achieves the properties of both metals [40]. Due to these properties, researchers have been interested in making a hybrid composite of Ag/Fe_2_O_3_ offering better catalytic, bactericidal, and bio-imaging properties [41,42,43]. To prepare metal nanoparticles, different techniques such as physicochemical, chemical, and green synthesis by using plant, algae, fungi, and bacterial extracts can be employed [44,45,46,47,48,49,50,51,52,53,54,55]. Plant-based NPs are highly active against different epidemic microbial diseases and are nontoxic to humans [56,57]. The easiest method to synthesize Ag/Fe_2_O_3_ nanocomposite by utilizing *Algaiia Monozyga* extract as a natural reducing and capping agent was studied [41]. Numerous experimenters have utilized *Rumix acetosa*, *Hordeum vulgar*, and *Azadiracta indica* in the green fabrication of Fe_2_O_3_NPs [58,59]. Leaf extract-mediated synthesis of Fe_2_O_3_NPs is gaining the attention of researchers because of its lower toxicity compared to other chemical reduction methods [28]. Due to their potential utility in targeted drug administration and magnetic hyperthermia cancer treatment, extensive study has been completed on the anticancer properties of Fe_2_O_3_NPs [60,61,62,63]. There have been very few reports of investigations on the creation of Ag/Fe_2_O_3_ NPs. According to Chen et al., several heteromers of Ag/Fe_2_O_3_ NPs may be synthesized and exhibit bactericidal properties [43]. It is hypothesized that a key factor in the antibacterial action of Ag/Fe_2_O_3_ NPs is the release of Ag^+^ ions [41]. Pathogens including bacteria and fungus, which are multi-drug-resistant pathogens, are more easily defeated by Ag/Fe_2_O_3_ NPs than by Ag NPs alone. To the best of our knowledge, this is the first publication to examine the biosynthesis of the Ag/Fe_2_O_3_ by *Buddleja lindleyana* leaves. The present study aims to study the green synthesis of nanocomposite Ag/Fe_2_O_3_ NPs-based safe natural *Buddleja lindleyana* leaves as a capping and reducing agent. Additionally, the impact of Ag/Fe_2_O_3_ NPs on harmful bacteria was investigated.

## 2. Materials and methods

### 2.1. Materials

*Buddleja lindleyana* leaves were collected in the UK. Fe_2_ (SO_4_)_3_·6H_2_O and silver nitrate (AgNO_3_, >99.98%) were purchased from Sigma-Aldrich. All aqueous solutions were prepared using distilled deionized (DI) water.

### 2.2. Biosynthesis of Ag/Fe_2_O_3_

Firstly, *Buddleja lindleyana* leaves were carefully washed with DI water to remove impurity and then dried for 2 days at room temperature. The *Buddleja lindleyana* leaves were then weighed out at 10 g and added to 100 mL of DI water. After being heated for 30 min at 90 °C, the mixture was filtered to filter out the broth [31]. The final extract was collected for further usage. For the synthesis of Ag/Fe_2_O_3_, a suitable aqueous solution of *Buddleja lindleyana* extract was gradually added into the mixture containing 1 g Fe(SO_4_)_3_·6H_2_O and 0.1 g silver nitrate (AgNO_3_) in an Erlenmeyer flask while stirring at 300 rpm with a magnetic stirrer at 70 °C for 3 h.

### 2.3. Characterization of Ag/Fe_2_O_3_

Preliminary characterization of the Ag/Fe_2_O_3_ prepared was carried out using UV-Vis spectroscopy. The measurement was carried out using a Jasco dual-beam spectrophotometer (model V-530, Tokyo, Japan) having an operational wavelength range of 300 to 800 nm. Thereafter, the formed pellet was used to identify functional groups present using FTIR spectroscopy. In addition, FTIR was employed to identify the functional groups present in the aqueous leaf extract that led to the formation of nanocomposite. For that, the dry leaf powder of *Buddleja lindleyana* was pelletized, and their FTIR (Jasco 460 plus, Tokyo, Japan) spectra were recorded between 4000 and 400 cm^−1^. UV-Vis spectroscopy was used to perform preliminary characterization of the Ag/Fe_2_O_3_ produced. With an operating wavelength range of 300 to 800 nm, the measurement was performed using a Jasco dual-beam spectrophotometer (model V-530, Tokyo, Japan). The produced pellet was then utilized to analyze the functional groups using FTIR spectroscopy. Additionally, FTIR was used to pinpoint the functional groups in the aqueous leaf extract that contributed to the creation of the nanocomposite. In order to do that, pellets of *Buddleja lindleyana*’s dry leaf powder were made, and their FTIR (Jasco, 460-plus, Tokyo, Japan) spectra were taken between 4000 and 400 cm^−1^. The FTIR spectra for *Buddleja lindleyana* extract and the spectrum for the aqueous extract of Ag/Fe_2_O_3_ were assembled. The size and morphological characterization of the synthesized and lyophilized Ag/Fe_2_O_3_ was assessed using transmission electron microscope (TEM). TEM (JEOL-1010, Tokyo, Japan) was used to describe Ag/Fe_2_O_3_ in order to determine their sizes and morphologies. The outcome was obtained by injecting the carbon-coated copper grid with Ag/Fe_2_O_3_ solution and placing it on a sample holder. Furthermore, the X-ray thin film diffraction measurement for the bio-reduced Ag/Fe_2_O_3_ was also performed using a Goniometer = PW3050/60 using Cu kα radiation at 40 kV and 25 °C. Subsequently, relevant X-ray patterns were obtained in the range of 20–80 °C. DLS (dynamic light scattering) particle sizing was employed to study the dispersion of Ag/Fe_2_O_3_ in colloidal utilizing the Zetasizer Nanosizer (Malvern-Instruments, Worcestershire, UK). For the particle size analysis, the Ag/Fe_2_O_3_ sample was re-suspended in purified water at 25 ppm and vortex-mixed to obtain a homogenous solution. A field emission scanning electron microscope (FESEM) (Quanta, 250-FEG, Taipei, Taiwan) was connected to an energy-dispersive X-ray analyzer (EDX, Unit). EDX and mapping were used to determine the surfaces of the prepared Ag/Fe_2_O_3_.

### 2.4. Assessment of the Inhibitory Activity of Ag/Fe_2_O_3_ against Pathogenic Microbes

The inhibitory activity of the Ag/Fe_2_O_3_ against bacteria and fungi was assessed using a well-diffusion test. Four bacteria including *B. subtilis*, *S. aureus*, *E. coli*, and *P. aeruginosa* were employed for the tests. Using the streaking method, test bacteria were inoculated into sterile Petri plates containing 20 mL of Mueller–Hinton. Sterile cork borer (6 mm) was used to prepare wells, and then, 200 µL of Ag/Fe_2_O_3_ at a concentration of 5 mg/mL was added in the well. The inoculated plates were kept in a refrigerator for 45 min to achieve adequate diffusion of Ag/Fe_2_O_3_, which was followed by incubation at 37 °C for 24 h for the bacteria test. The inhibition zone around each well was measured.

## 3. Results and Discussion

### 3.1. Synthesis of Ag/Fe_2_O_3_ Nanoparticles

**Figure 1:**
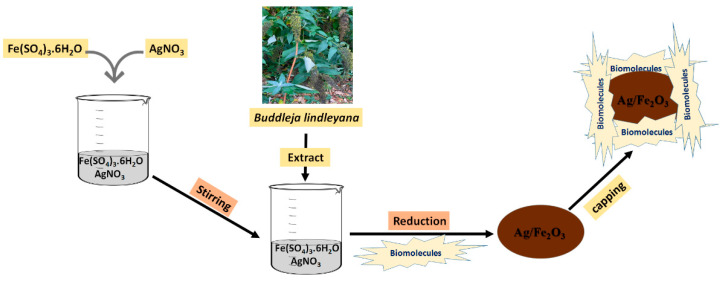
The mechanism of the reduction process of the Ag/Fe_2_O_3_ nanocomposite using *Buddleja lindleyana* extract.

One of the goals of this study was to use *Buddleja lindleyana* plant sources for the preparation of nanomaterials. The *Buddleja lindleyana* plant, which belongs to the Loganiaceae family, was chosen and used in this study for Ag/Fe_2_O_3_ synthesis. The main chemical components of *Buddleja lindleyana* include flavonoids, triterpenoids, phenylethaoids, and iridoid glycosides [64,65,66]. The primary idea behind this procedure was to develop a clean, environmentally friendly method of creating nanomaterials using *Buddleja lindleyana* extract, which has the ability to function as both a reducing and a stabilizing agent in the production of a Ag/Fe_2_O_3_ nanocomposite (Figure 1).

**Figure 2:**
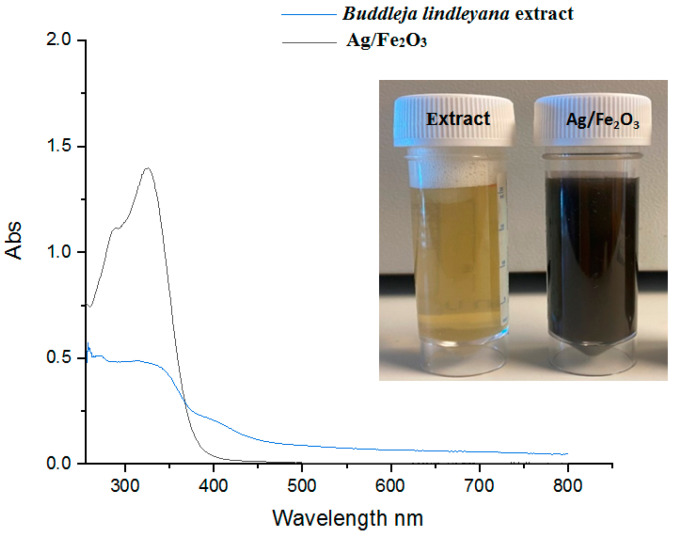
Color change and UV-Vis absorption spectra of Ag/Fe_2_O_3_ nanocomposite synthesis by *Buddleja lindleyana* extract.

The synthesis of Ag/Fe_2_O_3_ was conducted by adding Fe(SO_4_)_3_·6H_2_O and AgNO_3_ as a precursor to the *Buddleja lindleyana* leaf extract until a gradual change in the reaction color was observed. After 3 h of incubation at 70 °C, the color of the reaction mixture changed from pale green to dark brown (Figure 2). Such a change of color of the reaction mixture represents the formation of Ag/Fe_2_O_3_ by the *Buddleja lindleyana* extract. UV-Vis spectra of the Ag/Fe_2_O_3_ nanocomposite after 3 h were measured within the range of 300–800 nm and are presented in Figure 2. The strong peak formed at 320 nm, as visible in the UV-Vis spectra, was used to identify the formation of Ag/Fe_2_O_3_ nanocomposite. Similarly, UV-Vis studies of AgFeO_2_NPs by Berastegui et al. revealed high-level absorptions from 300 to 650 nm [67]. The presence of Ag/Fe_2_O_3_ nanocomposite caused the widening of absorbance at 320 nm (Figure 2).

**Figure 3:**
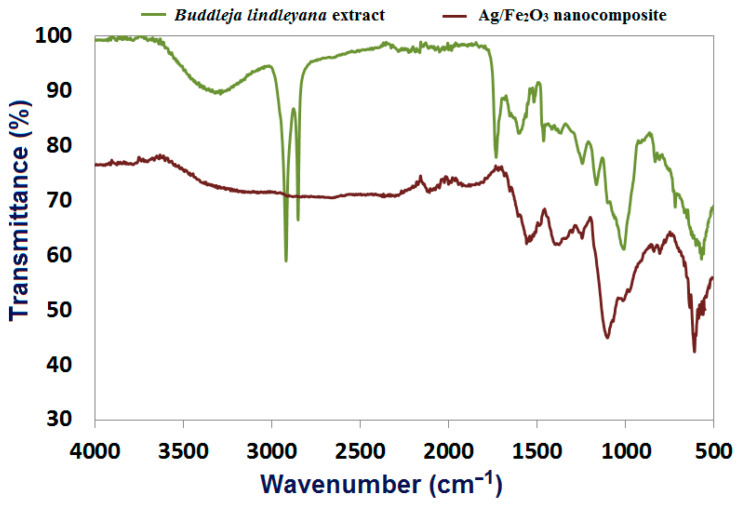
FTIR spectral analysis of *Buddleja lindleyana* extract and Ag/Fe_2_O_3_ nanocomposite.

FTIR spectroscopic analysis was used to investigate the structural features of the bioactive constituents in *Buddleja lindleyana* extract as well as the probable chemical alterations due to the formation of Ag/Fe_2_O_3_. As indicated in Figure 3, the bonding arrangements of *Buddleja lindleyana* were investigated using FTIR in the spectrum range of 400–4000 cm^−1^ (Figure 3). The H-bonded and -OH stretch vibrating of hydroxyl and phenolic groups of *Buddleja lindleyana* extract was found in a wide range 3367.9–3216.9 cm^−1^ [31]. The -CH group is responsible for the bands at 2918.5 cm^−1^ and 2850.6 cm^−1^. The presence of C-O of the ester group is indicated by the strong peak seen at 1728.7 cm^−1^. The existence of NH amine is shown by the peak at 1603.8 cm^−1^ [20]. The existence of functional groups such as carboxylic acid and ether is further confirmed by FTIR analysis, which provides a peak value of 1008 cm^−1^. The bioactive chemicals in *Buddleja lindleyana* extract are employed to convert ions into their appropriate metal forms. Furthermore, these phytochemicals have extremely reactive hydroxyl groups, which give hydrogen and so help to reduce the quantity of free radicals. The findings support the theory that these phytochemicals play a role in the bio-reduction process that leads to the production of nanomaterials [68,69]. According to the IR spectra of Ag/Fe_2_O_3_, the suppression of aliphatic molecules in Ag/Fe_2_O_3_ can be linked to redox changes of phytochemicals during Ag/Fe_2_O_3_ formation (Figure 3). Furthermore, IR studies revealed that chemical groups from the extract were bound to the Ag/Fe_2_O_3_ layer, indicating that *Buddleja lindleyana* extract worked as a stabilizer for the development of nanocomposite. The bending vibration of AgO and FeO interactions in Ag/Fe_2_O_3_ may explain the occurrence of peaks at 611.4 and 561.4 cm^−1^, respectively, in Figure 3.

### 3.2. Crystalline Structure of Ag/Fe_2_O_3_ Nanoparticles

**Figure 4:**
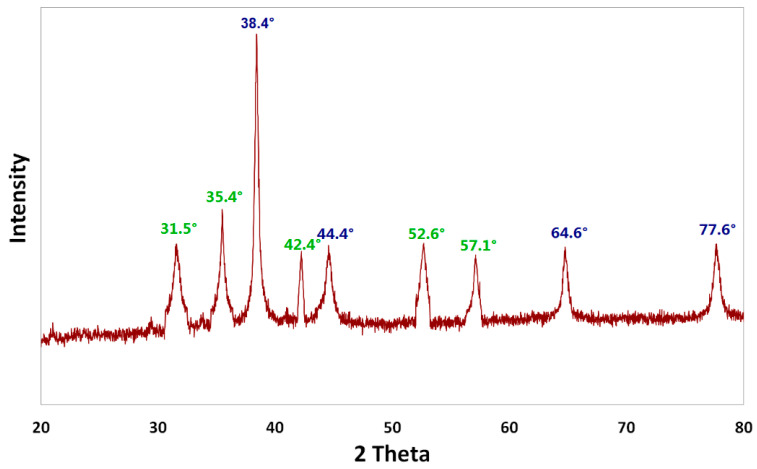
X-ray diffraction (XRD) of the Ag/Fe_2_O_3_ synthesized by *Buddleja lindleyana* extract.

The crystalline structure of the Ag/Fe_2_O_3_ NPs prepared was validated using XRD analysis, as shown in Figure 4. The primary strong angles in the diffractogram of the photosynthesized Ag/Fe_2_O_3_ were visible in the XRD patterns, showing that the Ag/Fe_2_O_3_ nanocomposite was crystallographic in nature. Bands at 31.5°, 35.4°, 38.4°, 42.4°, 44.4°, 52.6°, 57.1°, 64.6°, and 77.6° corresponded to Ag/Fe_2_O_3_ diffraction peaks. The values of silver appeared at the angles 38.4°, 44.4°, 64.6°, and 77.6° [20], while the peaks of the angles for iron oxide appeared at 31.5°, 35.4°, 42.4°, 52.6°, and 57.1° [31]. Therefore, the results clearly support the successful synthesis of nano Ag/Fe_2_O_3_. Diffraction patterns of the Ag/Fe_2_O_3_ nanocomposite did not show the presence of any contaminants, which verifies the purity of the Ag/Fe_2_O_3_ nanocomposite obtained.

**Figure 5:**
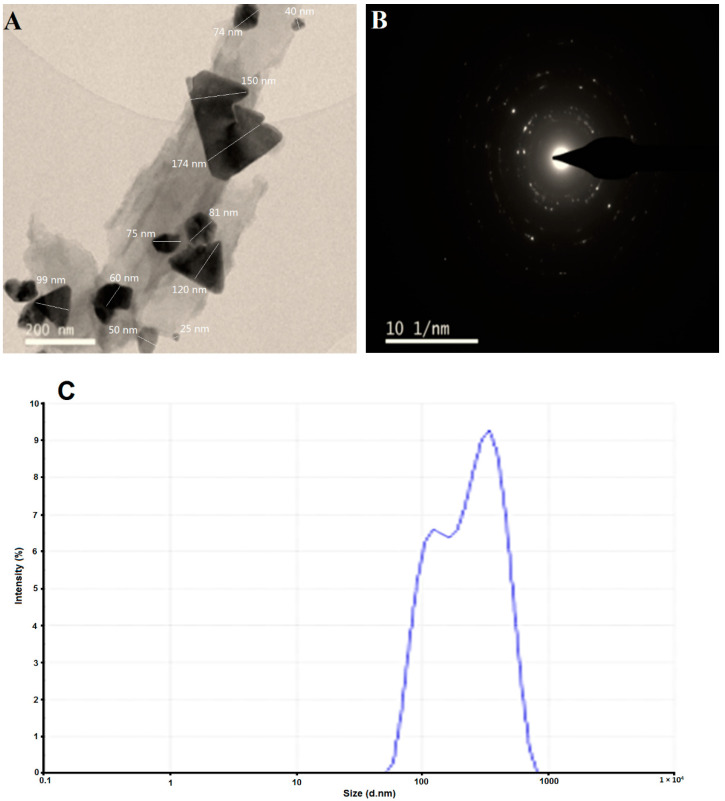
TEM image (**A**), SAED pattern (**B**), and DLS (**C**) of the synthesized Ag/Fe_2_O_3_ by *Buddleja lindleyana* extract.

The most widely used technique for determining the morphological features and sizes of nanostructures is the transmission electron microscope (TEM). Ag/Fe_2_O_3_ nanocomposites have been created in various forms such as triangular and spherical with an average size range of 25–174 nm, as seen in the TEM image (Figure 5A). These different forms are a representation of the majority of nanocomposites found in Ag and Fe_2_O_3_. The planes of the nanocomposite, as well as the degree of crystallinity of *Buddleja lindleyana* Ag/Fe_2_O_3_ particles, are shown by the bright circular areas in the SAED (selected area electron diffraction) pattern (Figure 5B). The SAED pattern’s ring patterns match the Fe_2_O_3_ NPs’ (104), (110), (113), (024), and (122) planes [70]. The SAED pattern further displays planes (100), (200), (220) and (311) that correspond to silver nanoparticles in addition to the aforementioned planes [71]. The average diameter of the Ag/Fe_2_O_3_ nanocomposite was determined using dynamic light scattering (DLS) analysis. The produced Ag/Fe_2_O_3_ nanocomposites were a poly-dispersed aggregate with an average diameter of 270.9 nm (Figure 5C). However, biomaterials deposited on the Ag/Fe_2_O_3_ surface by *Buddleja lindleyana*, such as organic compounds associated as stabilizers, as well as the metal core (Ag and Fe) of the Ag/Fe_2_O_3_ nanocomposite, alter the size determined by DLS [40,72].

**Figure 6:**
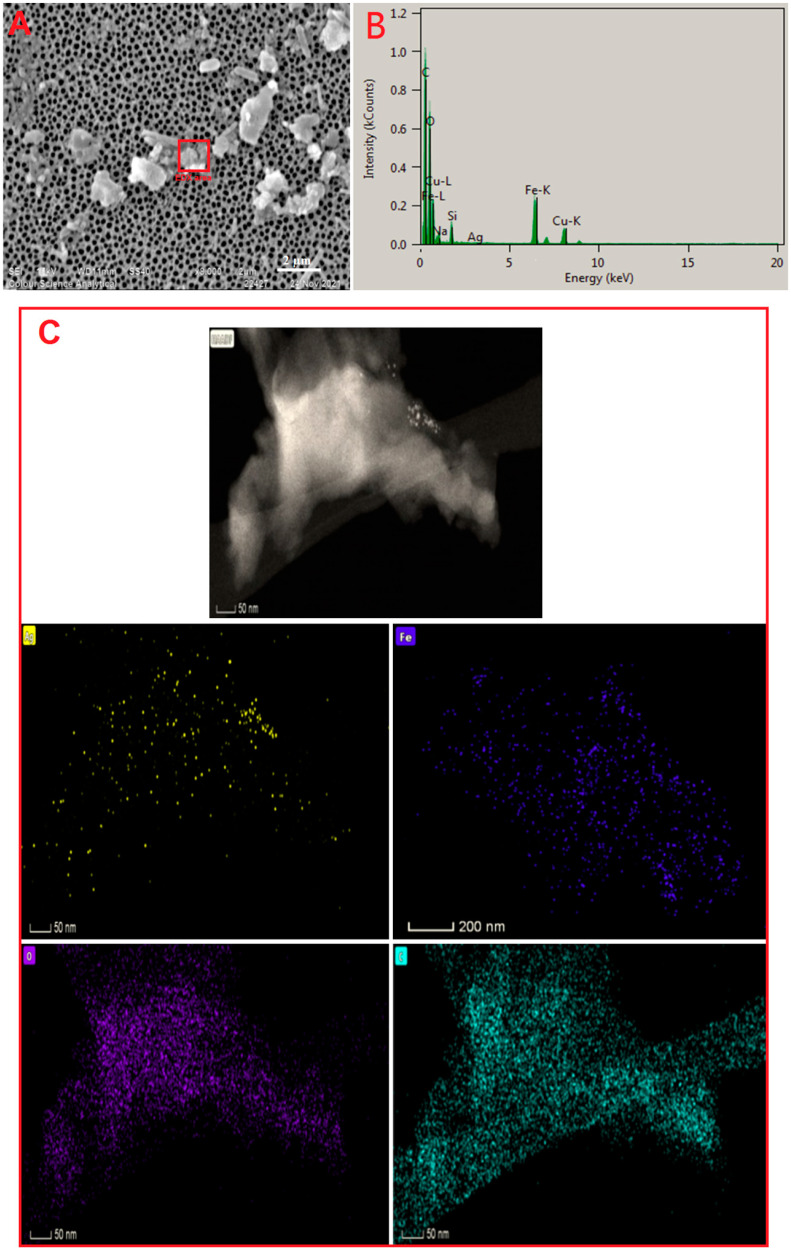
(**A**) SEM image, (**B**) EDX spectrum, and (**C**) element mapping of Ag/Fe_2_O_3_ nanocomposites.

SEM was used to analyze the morphology of Ag/Fe_2_O_3_ prepared using a simple and single-step process, as demonstrated in Figure 6A. The Ag/Fe_2_O_3_ seemed to have a mono-dispersed and aggregation-free micrograph. The EDX spectra revealed the existence of several well-defined peaks in the Ag/Fe_2_O_3_ nanocomposite that were due to silver, iron, oxygen, and carbon components (Figure 6B). Furthermore, EDX spectra revealed the production of a very pure Ag/Fe_2_O_3_ nanocomposite with no additional impurity-related peaks. The SEM image and the EDX spectra of the nanocomposite prepared revealed that the Ag/Fe_2_O_3_ nanostructures were well distributed in the *Buddleja lindleyana* extract [73]. In addition, elemental EDX mapping was used to determine the Ag and Fe elemental distribution (spatial/lateral) of the Ag/Fe_2_O_3_ nanocomposite prepared. The mapping of Fe, Ag, O, and C elements can be seen in the picture (Figure 6C). Both Fe and Ag were uniformly distributed throughout the Ag/Fe_2_O_3_ sample, according to the EDX elemental analysis shown in the Ag, Fe, O, and C element mapping images of Ag/Fe_2_O_3_ (Figure 6) [41].

### 3.3. Antimicrobial Activity

**Figure 7:**
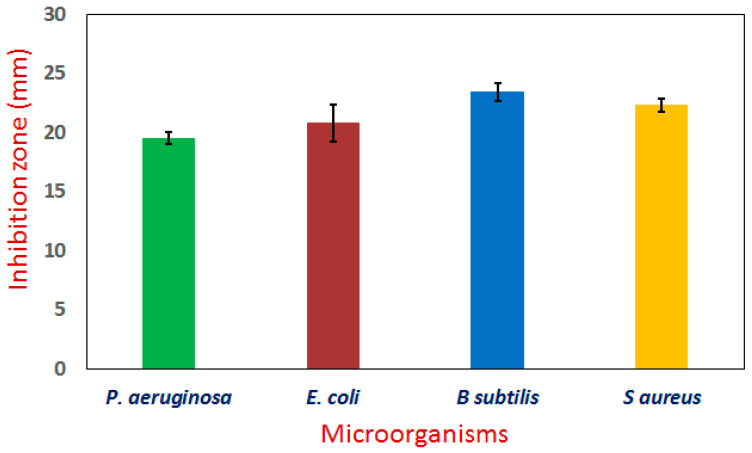
Antimicrobial activity of the synthesized Ag/Fe_2_O_3_ by *Buddleja lindleyana* extract.

Recently, several pathogenic microorganisms have developed resistance to currently available commercial antibiotic agents and also caused adverse impact on human health. Thus, new active and safe antimicrobial agents are required. Because nanomaterials have antibacterial capabilities, they have recently received attention [74,75]. According to Figure 7, the well diffusion technique was used in this investigation to evaluate the Ag/Fe_2_O_3_ nanocomposite’s antibacterial efficacy against human bacterial pathogens (*S aureus*, *E. coli*, *B subtilis*, and *P. aeruginosa*). The maximal Ag/Fe_2_O_3_ dose (5 g/mL) showed a high level of inhibitory activity against the pathogens *B. subtilis*, *S. aureus*, *E. coli*, and *P. aeruginosa* with inhibition zones of 23.4 ± 0.75, 22.3 ± 0.57, 20.8 ± 1.6 and 19.5 ± 0.5 mm, respectively (Figure 7). A previous study showed that silver particles have a good antimicrobial effect on harmful bacteria, whether they are Gram-positive or Gram-negative bacteria [43]. The antibacterial activity of Ag/Fe_2_O_3_ nanocomposite was investigated in earlier investigations, and it showed good activity against Gram-negative and Gram-positive bacteria [34,40,76]. Ag/Fe_2_O_3_ has an antibacterial impact on bacteria because it damages cell walls, disrupts structural proteins, inactivates enzymes, inhibits electron transport chains, damages nucleic acids (DNA), and causes oxidative stress brought on by the generation of reactive oxygen species (ROS) [41,72,77]. As a result, Ag/Fe_2_O_3_ has emerged as a viable antibacterial treatment option and is useful in the medicinal field.

## 4. Conclusions

*Buddleja lindleyana* extract was used for the phytosynthesis of Ag/Fe_2_O_3_NP through an eco-friendly route. The fabricated Ag/Fe_2_O_3_ nanocomposites were characterized using UV-Vis, FTIR, XRD, TEM, DLS and SEM-EDX analyses. The Ag/Fe_2_O_3_ nanocomposites obtained are triangular–spheroidal and exhibit a high absorbance band around 320 nm. According to the observation made from the FTIR analysis, it is possible to conclude that the compounds present in *Buddleja lindleyana*’s extract play an important role in the reduction and stabilization of nanocomposites. The Ag/Fe_2_O_3_NPs obtained are stable in colloidal solution. Results also revealed that the Ag/Fe_2_O_3_NP nanocomposites fabricated have outstanding antimicrobial activity against pathogenic strains (*S aureus*, *E. coli*, *B subtilis*, and *P. aeruginosa*). It was found that the antimicrobial activity of the Ag/Fe_2_O_3_ nanocomposites is dependent on the structure of the nanocomposites, which have significant activity against all bacterial strains tested. Finally, the Ag/Fe_2_O_3_ nanocomposites biosynthesized using the extract of *Buddleja lindleyana* were also shown to have antibacterial properties, making them promising materials for usage in the medical field.

## Data Availability

The data used to support the findings of this study are available from the corresponding author upon request.
